# Chemical Modification and Foam Processing of Polylactide (PLA)

**DOI:** 10.3390/polym11020306

**Published:** 2019-02-12

**Authors:** Tobias Standau, Chunjing Zhao, Svenja Murillo Castellón, Christian Bonten, Volker Altstädt

**Affiliations:** 1 Depatment of Polymer Engineering, University Bayreuth, Universitätsstraße 30, 95447 Bayreuth, Germany; tobias.standau@uni-bayreuth.de (T.S.); chunjing.zhao@uni-bayreuth.de (C.Z.); 2 Institut für Kunststofftechnik, University of Stuttgart, Pfaffenwaldring 32, 70569 Stuttgart, Germany; svenja.murillo.castellon@ikt.uni-stuttgart.de (S.M.C.); christian.bonten@ikt.uni-stuttgart.de (C.B.); 3 Bavarian Polymer Institute and Bayreuth Institute of Macromolecular Research, University of Bayreuth, Universitätsstraße 30, 95447 Bayreuth, Germany

**Keywords:** polylactide (PLA), biofoams, chemical modification, foam extrusion, batch foaming, foam injection molding, bead foaming, rheology, crystallization, density reduction

## Abstract

Polylactide (PLA) is known as one of the most promising biopolymers as it is derived from renewable feedstock and can be biodegraded. During the last two decades, it moved more and more into the focus of scientific research and industrial use. It is even considered as a suitable replacement for standard petroleum-based polymers, such as polystyrene (PS), which can be found in a wide range of applications—amongst others in foams for packaging and insulation applications—but cause strong environmental issues. PLA has comparable mechanical properties to PS. However, the lack of melt strength is often referred to as a drawback for most foaming processes. One way to overcome this issue is the incorporation of chemical modifiers which can induce chain extension, branching, or cross-linking. As such, a wide variety of substances were studied in the literature. This work should give an overview of the most commonly used chemical modifiers and their effects on rheological, thermal, and foaming behavior. Therefore, this review article summarizes the research conducted on neat and chemically modified PLA foamed with the conventional foaming methods (i.e., batch foaming, foam extrusion, foam injection molding, and bead foaming).

## 1. Introduction

Polylactide is an aliphatic polyester that can be derived from renewable resources. An early description of its synthesis from lactide was given by Carothers et al. [1] in 1932, followed by a patent from DuPont [2] in 1954. Because the monomer lactic acid is chiral, two optical isomers exist. The more common isomer is l-(+)-lactic acid, or (*S*)-lactic acid. The d-(−)-lactic acid or (*R*)-lactic acid is the rarely obtained isomer. Consequently, for the polymer, it can also be differentiated between l- and d-polylactide (PLA). Usually, commercial grades are a mixture of l- and d-PLA, but l-PLA can be found predominantly. Depending on the ratio, the properties can vary significantly. Also, the pure co-monomers, i.e., pure l-PLA and d-PLA, can be found commercially (for example, from Corbion/Purac B.V.).

**Figure 1:**
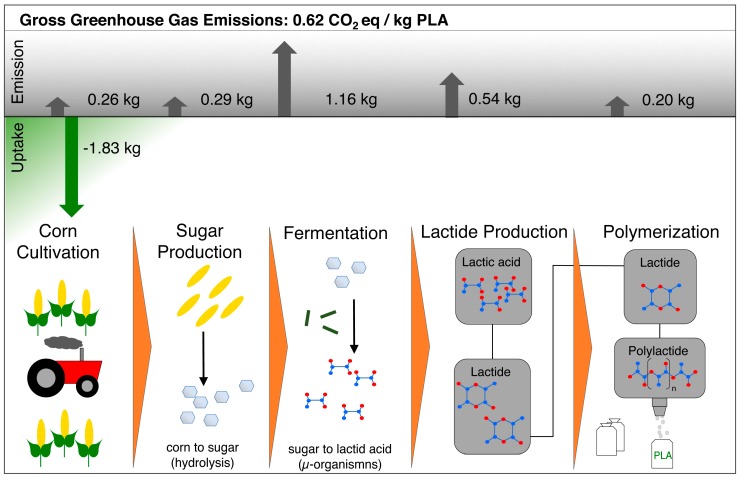
Steps of polylactide (PLA) production with greenhouse gas uptake and emissions for 1 kg of PLA (based on the data of Reference [4]).

Currently, most PLA is derived from feedstock corn. However, other plants delivering carbohydrates are possible to use in the industrial production of PLA, such as potatoes, cassava, rice, wheat [3], or sugar cane and sugar beets [4]. A description of the large-scale production of PLA was given by Lunt [5] and Auras et al. [6]. In Figure 1 a sketch of how the corn is transformed into PLA is shown. Additionally, the carbon footprint for the single steps is given. During the growth of the corn, CO_2_ is taken up by the plants from the atmosphere, which constitutes the green characteristics of PLA at the end. The corn delivers sugar (dextrose) which is converted into lactic acid in a fermentation process by bacteria. An overview of suitable microorganisms and their lactic acid yield was given by Nampoothiri et al. [7]. This fermentation mainly delivers the l-isomer [8]. The polymerization is usually done with lactide, the dimer of lactic acid. Because of the high amount of CO_2_ that is taken up during plant growth, the gross greenhouse emissions of PLA are compensated for down to 0.62 kg CO_2_ equivalent per kg polymer, according to the manufacturer NatureWorks LLC [4]. Here, other factors that also have an impact on the environment such as the use of fertilizers, pesticides, energy, etc. are taken into account [9]. Similar values (0.5 to 0.8 kg CO_2_ eq/kg polymer) can be found in a publication from Groot and Borén about PLA derived from sugar cane of the company Purac B.V. [10].

Biopolymers comprise polymers that are either (i) bio-based and biodegradable or (ii) bio-based but non-degradable (so-called drop-ins), as well as (iii) those fossil-based polymers that are biodegradable. Currently, the drop-ins hold the biggest market share for biopolymers, i.e., bio-PET (e.g., polyethylene terephthalate from SCG Chemicals Co., Ltd.), bio-PA (e.g., polyamide from Evonik Industries AG) bio-PE, and bio-PP (e.g., polyethylene and polypropylene both from Braskem S.A.). PLA and starch are the most important biopolymers that are bio-based and biodegradable, each with a global production of roughly 200,000 t/y [11]. PLA is well known as the most promising bio-based and biodegradable polymer with properties and processability that come close to those of (i) PS [6,12,13] and (ii) polyvinyl chloride (PVC), PE, or PP when plasticized with its own monomers [12]. Up to now, PLA can be found in a lot of products such as disposable cups, dishes, cutlery, bottles, wovens, and electronics [3,14]. Furthermore, because of its biocompatibility and biodegradability, it is well suitable for medical applications [8,15]. An initial work on foaming of PLA was conducted with the batch foam method in 1996 by Mooney et al. [16]. The foam extrusion of PLA (in combination with starch) is another early approach, which was carried out by Fang et al. [17] in 2000. Since then, a lot of research was done on foaming of PLA and, in the last few years, PLA foams were even established commercially, such as thermoformed extrusion sheet foams from Sealed Air Corp. (Cryovac NatureTRAY^TM^) [18], as well as bead foams from the companies Synbra Technology BV (BioFoam^®^) [19] and BASF SE (ecovio^®^EA) [20,21].

Even though prices for PLA decreased significantly below $2000/ton over the last decade, the standard polymers are still lower priced, as polystyrene, for example, can be purchased for less than $1000/ton. Thus, PLA is not fully economically competitive compared to the fossil-based polymers.

PLA used for foam research is purchased from different companies; amongst others, Biomer [22], Mitsui Chemicals [23,24], Corbion/Purac Biochem B.V. [25], and Unitika Ltd. [26,27,28,29] can be found as producers. However, the biggest supplier for PLA is NatureWorks LLC with a capacity of 150,000 t/y [4]. In Table 1, the literature about foams produced with PLA grades from NatureWorks LLC is summarized, giving information about the grade and its internationally recommended processing method by the supplier, d-content, the foaming method, and whether or not chemical modifiers were used. 

In literature, the low melt strength of PLA is often designated as the main challenge for foaming PLA [119]. Nevertheless, among the numerous works about foaming PLA, only a handful of publications quantify the melt strength at all [52,59,109,120,121]. To enhance the foamability of PLA, several strategies as summarized by Nofar and Park [122], could be followed; these include (i) the introduction of chemical modifications such as chain extenders to increase molecular weight and/or introduce branched or even cross-linked structures, (ii) the modification of d-ratio, (iii) the addition of additives, and (iv) the enhancement of the slow crystallization kinetics. The approach of chemical (melt) modification was followed by a lot of research groups and a huge variety of substances to modify PLA were described, such as multifunctional epoxy chain extenders [68,121,123] (Joncryl^®^ from BASF SE is the most commonly used commercial product), peroxides (lauroyl peroxide [109,120], dicumyl peroxide [121,124,125]), maleic anhydride [109,121], oxazoline [109,121], and many more. The next section describes substances that are used for melt modification and how they affect the properties and foam processing of PLA.

## 2. Chemical Modifications

Conventional chemical modifications of PLA to increase molecular weight (MW) and/or to introduce extended, branched, or cross-linked structures include (i) ring-opening copolymerization, (ii) reactions of low-MW prepolymers of PLA with chain extenders, such as epoxy [126], diisocyanate [127,128], and oxazoline [129], and (iii) post-polymerization reactions, including melt modification and radiation treatment [130]. Here, the melt modification by reactive extrusion is of high relevance, because it is a cost-effective and convenient method to adjust the desired properties, as the suited chemical modifier(s) are directly added into PLA melt in the reactive extrusion process (in situ). Thus, the modification of commercial grades can be done individually in a short time at different scales, giving more flexibility [131,132]. Currently, melt modification by reactive extrusion is the mainly studied method in engineering research and is widely applied in industry. Therefore, only melt modification by reactive extrusion is discussed in this review.

During processing, especially at high temperatures, PLA undergoes degradation, such as hydrolysis, backbiting, or depolymerization, leading to undesirable MW reduction by random chain scission [133]. However, chemical modifiers enable the relinking of polymer chains, thereby increasing the MW of the polymer again [134]. The reactive extrusion of PLA with chemical modifiers is a complex process, in which both polymer chain scission and chain recombination take place [135,136]. Additionally, secondary reactions, such as transesterification [126,137] or homopolymerization [138,139] could happen. Therefore, chain extension, branching, and/or cross-linking of PLA depend on various factors such as the type and concentration of modifier, processing temperature, and reaction time. There are different branched structures reported for modified PLA such as star-shaped, comb-shaped, dendritic/hyperbranched, H-shaped, long-chain branched, or dumbbell-shaped, exhibiting different properties [140]. In general, the chain recombination induced by chemical modification can be distinguished between two kinds of reactions, which are—as discussed below—(i) reactive functional group reaction, and (ii) free-radical reaction.

### 2.1. Functional Group Reaction

For functional group reaction, the chemical modifier is normally referred to as a chain extender (CE). The reactive groups of CE, such as carboxylic, hydroxyl, epoxy, isocyanate, amine groups, etc., can react with the carboxyl and hydroxyl groups of PLA [36,123,141]. Bifunctional CEs only lead to a chain-extended linear PLA as it will couple exactly two end groups [142]. For multifunctional CEs, chain-branching could happen. Here, the degree of branching depends on the functionality and concentration of the CE [123,135]. However, chain extension was reported to be dominant because chain-branching requires higher activation energy and a longer reaction time [36]. In Table 2, commonly used CEs for melt modification of PLA are listed.

#### 2.1.1. Epoxide

Epoxide groups can react with carboxyl and hydroxyl chain end groups of PLA through the ring-opening reaction, forming covalent bonds [123]. The commercial product Joncryl^®^ from BASF SE, a multifunctional styrene–acrylic–epoxy-based random oligomer, is the most common used CE for PLA due to its high functionality and chain-extending efficiency [142]. Alternatively, masterbatches containing Joncryl are produced by Clariant under the tradename CESA-Extend^®^ [134]. Since Joncryl has multiple reactive sites, several PLA chains could be chemically connected by one CE molecule, resulting in chain extension, branching, and/or cross-linking [101]. Some researchers reported a comb-like chain structure of Joncryl-modified PLA [139,143,177] 

#### 2.1.2. Diisocyanate

Diisocyanates, such as 1,6-hexamethylene diisocyanate (HDI) [178] and 4,4-methylene diphenyl diisocyanate (MDI) [127,159], are more often used in the solution chain extension process for PLA prepolymers. However, melt modification with diisocyanates was also studied. The isocyanate can react with both carboxyl and hydroxyl groups of PLA to form ester–urethane linkages. The reactivity of isocyanate with hydroxyl groups is much higher than that with carboxyl groups [158]. Furthermore, isocyanates can also act as a coupling agent between polymer blends, such as PLA and starch [179,180], PLA and poly(ether-*b*-amide) (PEBA) [160]. In the work of Di et al. [67], 1,4-butane diisocyanate (BDI) incorporated with 1,4-butanediol was applied for PLA in order to improve the foamability through chain extension and cross-linking.

#### 2.1.3. Dianhydride

Pyromellitic dianhydride (PMDA) is a chain extender reacting with hydroxyl groups of PLA via a ring-opening reaction of the anhydride. Liu et al. [162] increased the melt strength of PLA by incorporating PMDA. It was reported by Gu et al. that the reactivity of PMDA with hydroxyl groups is relatively low [143]. Therefore, the combination of PMDA with other chemical modifiers is an efficient way to enhance the reactivity and to promote branching. The authors used PMDA with trimethylolpropane tris(2-methyl-1-aziridinepropionate) (TTMAP), which has a high reactivity with the carboxyl group of PLA, and generated long-chain branching (LCB). Furthermore, according to the work of Liu et al. [36,164], by adding both PMDA and triglycidyl isocyanurate (TGIC), PLA with various chain structures including linear chains, star-like structures with three arms, and tree-like structures were obtained. PMDA was also used with oxazoline to obtain long-chain branched PLA [164]. However, PMDA is hygroscopic and can absorb moisture, causing hydrolysis of dianhydride groups to acid groups, which favors the thermal degradation of PLA [101].

#### 2.1.4. Oxazoline

Oxazolines is mostly used in solution reaction with PLA oligomers [129,181,182] and only limited research was based on the reactive extrusion process. Oxazoline reacts with carboxyl groups of PLA through a ring-opening reaction, and chain extension is achieved by the ester–amide linkages. It was observed that oxazoline-modified PLA exhibits improved melt strength [162]. Yu et al. [109] produced oxazoline-modified PLA batch foams with uniform cell size distribution and almost no cell rupture, which was attributed to the cell stabilization by improved melt strength.

#### 2.1.5. Carbodiimide

Carbodiimides (CDI) and mainly polycarbodiimides (PCDI) are CEs which react with carboxyl and hydroxyl groups of PLA [167]. Noteworthily, CDI is more reactive with carboxyl groups than with hydroxyl groups [166,168]. In addition to the chain extension reaction with PLA, CDI can also react with moisture/water to reduce hydrolysis. Therefore, it is an important stabilizer for PLA. The stabilizing effect of bis(2,6-diisopropylphenyl) carbodiimide (BDICDI) was studied by Stloukal et al. [166] and Holcapkova et al. [170], showing fewer chain scissions of ester bonds during abiotic hydrolysis and improved thermal stability by scavenging free carboxylic groups and water molecules. Najafi et al. [149] compared the effect of PCDI and epoxide-based CE on PLA and concluded that PCDI was less efficient in increasing MW and viscosity, and only extended linear chains were obtained. 

#### 2.1.6. Phosphite

Phosphite can react with the hydroxyl group and carboxyl group of PLA and, therefore, extend polymer chains [171,175]. Tris(nony1-phenyl) phosphite (TNPP) is a commonly used phosphite-based stabilizer for PLA, which showed excellent stabilizing effects on the MW of PLA at different temperatures [174]. Lehermeier and Dorgan [173] found that only 0.35 wt % of TNPP was enough to stabilize PLA during rheological measurement up to 200 °C with negligible degradation. Similar to the effect of PCDI, the addition of TNPP helped PLA chain relinking but did not lead to formation of branches [149]. In addition to TNPP, other phosphite-based CEs, such as triphenylphosphite (TPP), were also reported by Meng et al. [175] to stabilize PLA effectively by chain extension. In a further study [176], it was revealed that a concentration of 2 wt % TPP is most effective to increase the MW by a factor of 1.5 compared to virgin PLA, but higher concentrations would result in lower MW, as the by-products of TPP can cause more chemical degradation.

### 2.2. Free-Radical Reaction

Compared to chain-extender-modified PLA, PLA modified by free-radical reaction exhibits less controlled chain structures due to the randomness of free-radical reactions [36,143]. 

#### 2.2.1. Peroxide

Peroxide acts as a free-radical initiator in the reactive extrusion process to induce cross-linking of PLA [125,183,184]. It can be used solely or together with other chemical modifiers. Firstly, peroxide decomposes into primary radicals (RO·) when exposed to heat. Subsequently, hydrogen abstraction happens with the primary radicals, which allows the so-formed PLA macroradicals (PLA·) to recombine with each other and form C–C bonds [125,183]. The first step, namely free alkoxy radical formation, is the determining step for the degree of cross-linking [185]. Like the reactive extrusion process with CEs, in the presence of the radicals, PLA also undergoes both chain combination (i.e., branching and/or cross-linking) and chain scission [137,183,186]. Hence, the reaction conditions and peroxide concentration are crucial for keeping the optimum balance.

There are different types of peroxide providing a broad range of reactivity, such as lauroyl peroxide [109,120], diacyl peroxide, peroxyester, diperoxyketal, dialkyl peroxide, hydroperoxide, ketoneperoxide, and peroxydicarbonate [184,187]. The effect of the type of peroxide on cross-linking of PLA in reactive extrusion was studied by Takamura et al. [184]. Peroxides with a higher reaction rate, which result in a higher decomposition rate and shorter lifetime, only induced partial cross-linking of PLA, since the decomposition of peroxide took place very fast and PLA was still not fully molten. On the contrary, peroxides with a slower decomposition rate, for which the lifetime is relatively close to the residence time of extrusion, decomposed uniformly on molten PLA resulting in uniform cross-linking [184,188]. The decomposition rate of peroxides is also dependent on the processing temperature. At high temperature, peroxide decomposes faster, leading to a reduced lifetime and an increased radical concentration [189].

Dicumyl peroxide (DCP), is a monofunctional ditertiary alkyl peroxide, exhibiting a relatively slow decomposition rate and high hydrogen abstraction ability. Thus, DCP was applied extensively as cross-linking agent for PLA. Liu et al. [36] reported that PLA modified by DCP consisted of linear chains and a small amount of comb-like chains with about three arms, which is in good agreement with the finding of You et al. [163]. It was found by Södergård [190] that branching was dominant in PLA when DCP concentration was lower than 0.25 wt %, while, above this concentration, significant cross-linking could be noted. In addition to branching and cross-linking during the reactive extrusion, low-MW side products were formed following decomposition and degradation, which acted as plasticizers in the process [125,191]. This effect got more pronounced with higher DCP content. The same phenomenon was found by Huang et al. [124] and Wei et al. [183]. Additional multifunctional coagents, such as pentaerythritol triacrylate (PETA) [163], triallyl isocyanurate (TAIC) [192], and triallyl trimesate (TAM) [193], can be used together with peroxide to facilitate LCB formation by grafting onto the PLA backbone. PETA was suggested to be an efficient coagent with DCP by introducing more branched structures and reducing PLA degradation [163]. Yang et al. [192] found that cross-linked structures of PLA became significantly evident in the presence of DCP and a small amount of TAIC from 0.15 wt.% to 3 wt %.

In addition to being applied to produce branched and cross-linked PLA, peroxides also act as compatibilizers in PLA-based blends, such as PLA with polybutylene succinate (PBS) [144], polyhydroxybutyrate (PHB) [194], polybutylene adipate terephthalate (PBAT) [186], and natural rubber [124]. Due to the free-radical reaction triggered by peroxides, branching and cross-linking between the polymer chains in blends can be formed, resulting in improved phase compatibilization and interfacial adhesion, thus enhancing the mechanical properties.

#### 2.2.2. Grafting

Grafting is another approach to modify PLA. In the presence of free radicals, monomers and polymers can be grafted onto the backbone of PLA chains. Maleic anhydride (MA) is one of the most extensively used grafting pendants due to its good chemical reactivity, low toxicity, and low potential for homopolymerization under free-radical grafting conditions [132]. MA is highly reactive with PLA radicals initiated by peroxide, such as 2,5-bis(tert-butylperoxy)-2,5 dimethylhexane [195,196,197,198], DCP [199,200], and dibenzoylperoxide (DBPO) [201]. PLA radicals can react either with grafting monomers or undergo chain scission [197,199]. The properties of grafted PLA (PLA-g-MA) depend on concentrations and the ratio of peroxide and MA [132]. Increasing the peroxide concentration results in an increase in the number of free radicals and, therefore, the grafting of MA. It was noted that, in the absence of peroxide, MA showed no effect on the MW of PLA [121,196]. 

Free-radical-initiated grafting with MA was used to improve the viscosity and melt strength of polypropylene (PP) [202]. However, for PLA, grafting with MA resulted in decreased MW and viscosity [47,137,162,196,197,198,200,201]. It was reported by Yu et al. that the melt strength of PLA-g-MA decreased and consequently leaded to cell rupture during foaming [109]. Therefore, grafting with MA was mainly reported as a compatibilization approach for PLA blends. Improved interfacial adhesion between PLA and starch was observed via the reaction of MA with hydroxyl groups on the surface of starch [47,195,196,198].

## 3. Rheological Behavior

There are different factors determining the rheological properties of PLA, such as MW, polydispersity index (PDI), molecular structure, chain length, number of entanglements, etc. The melt of unmodified linear PLA behaves viscoelastically and exhibits the typical rheological properties of a non-Newtonian fluid, i.e., a Newtonian region at low frequencies followed by shear thinning and fast chain relaxation in shear flow and a linear response over time until constant in elongational flow [121,203]. Furthermore, linear PLA possesses low melt elasticity and low melt strength, which are disadvantageous for the foaming process, leading to cell rupture and coalescence during the cell growth process, resulting in inhomogeneous cell morphology and/or a low foam expansion ratio [64]. As described above, chemical modification of PLA leads to extended chains, short/long-chain branching, and cross-linking. Below, the change in the rheological properties (shear and elongation) as a result of increased MW, and the formation of non-linear structures that can be expected after chemical modification are described:

### 3.1. Shear Rheology

#### 3.1.1. Increased Zero Shear Viscosity

The zero shear viscosity $\eta_{0}$ depends on the average MW and the number of entanglements between two branching points [121,204]. Consequently, modified PLA with higher MW possesses an increased $\eta_{0}$. On the other hand, for comparable MW, it was found that $\eta_{0}$ decreased with the increase in the degree of branching, which could be explained by the decrease in hydrodynamic volume, traceable in a change of such as molecular radius of gyration, *R*g [121,203]. For instance, Dorgan et al. [203] also found that, for PLA with similar MW, the $\eta_{0}$ of star-shaped PLA with six arms was lower than that with four arms. In short, $\eta_{0}$ of PLA with a lower degree of LCB, such as comb- or star-like structures, will be higher, and $\eta_{0}$ of highly long-chain branched PLA, such as tree-like PLA, will be lower compared to their linear counterparts [121]. In another work of Lehermeier et al. [173], it was shown for linear PLA blended with branched PLA that $\eta_{0}$ increases with the amount of branched PLA. In addition to the change in $\eta_{0}$, the Newtonian plateau of modified PLA is also noticeably shortened and shifted to a lower angular frequency [123,147,205]. 

#### 3.1.2. Pronounced Shear Thinning Effect

In shear experiments, the shear thinning follows the Newtonian region. The shear thinning of modified PLA become more pronounced than that of the linear PLA [139,143,145,147,148,150,183], which is attributed to the additional effect from entanglement density reduction at higher shear rates.

#### 3.1.3. Increased Shear Viscosity

Moreover, complex viscosity as a function of angular frequency and steady viscosity as a function of shear rate are also increased due to the increased number of chain entanglements in modified PLA, which is typically correlated to the concentration of chemical modifier [123,124,139,145,147,148,150,183]. Some researchers [123,150,173,174] revealed that the Cox–Merz rule [206], being valid for linear PLA in a large range of shear rates and frequencies, was only applicable for branched PLA in a limited range and, to a certain extent, indicated high branched content or melt inhomogeneity such as cross-linking/gelation.

#### 3.1.4. Improved Melt Elasticity

For linear PLA, melt elasticity increases with MW [205]. It can also be improved with a branched structure and a high degree of chain entanglement, which is beneficial for foaming processes, as larger expansions are noticeable [31,67]. The enhancement of melt elasticity can be identified by the increase in storage modulus (G’) [52,67,135] or recoverable shear compliance [173]. 

#### 3.1.5. Enhanced Melt Stability

As PLA is a polymer which can easily degrade during processing at elevated temperatures, the melt stability is another important property. Melt stability of PLA can be studied by dynamical time sweep measurements [101,121,123,150,168,174] usually revealing an early onset of degradation with decreasing viscosity after a short time. On the contrary, modified structures help stabilize PLA at higher temperatures to a large extent, which means the onset of the degradation can be delayed and degradation kinetics can be reduced. 

### 3.2. Elongational Rheology

#### 3.2.1. Improved Melt Strength

Low melt strength, which is the major negative effect for foaming of PLA, can be overcome using chemical modification [119]. The few works [52,59,109] that quantified the melt strength showed that it can be raised with increased MW and an increased degree of branching [120,121]. For example, the work of Dean et al. [120] reported how the melt strength depends on the concentration of the added modifier lauroyl peroxide. Here, the addition of 1 wt.% led to a threefold higher melt strength. Some authors correlated the higher melt strength with improved foaming performance of modified PLA [51,59,109].

#### 3.2.2. Strain Hardening

It is well known that linear PLA does not exhibit strain hardening under extension [36,123]. However, the branching induced by chemical modification can lead to strain hardening, which is an increase in the extensional viscosity above the linear viscoelastic curve [207]. It is supposed to be advantageous for processes in which polymer melt will be stretched and melt strengthening is desired, such as spinning, film casting, blow molding, and foaming [81]. Strain hardening of branched polymers occurs due to chain stretching when polymer melt undergoes extensional deformation [208]. Therefore, the higher the branching degree is, the more significant the strain hardening will be. It was reported that branched chains with more than two branching points would exhibit evident strain hardening [36]. Gu et al. [177] reported that, although star-shaped PLA with three arms only exhibited enhanced elongational viscosity, no strain hardening effect was observed, due to the low LCB degree. Palade et al. [174] showed PLA with strain hardening as a result of high-molecular-weight tails. Also, via the incorporation of multifunctional epoxy-based CE with PLA, strain hardening was induced, as can be seen in the work of Corre et al. [123].

## 4. Crystallization Behavior

PLA can be found in an amorphous or semi-crystalline state. This depends on the stereo chemistry (i.e., ratio of l- and d-isomer) and the thermal history, as well described in the review of Lim et al. [119]. Commercial grades are usually l-rich PLA with d-lactide as the minor unit [209]. However, with increasing d-lactide content, melting temperature (*T*_m_), glass transition temperature (*T*_g_), and crystallinity decrease due to the higher amorphous amount and crystal disruption by d-lactide [172,210,211,212]. High d-lactide content results in a completely amorphous PLA. Different values for the d-lactide content, above which the PLA is amorphous, exist, i.e., above 15% [48], above 10 to 12% [209], above 8% [119], and ranging between 7 and 50% [118]. Crystalline PLA has higher heat resistance but reduced degradation rate [209]. 

Four crystal structures ($\alpha ,\alpha^{\prime },\;\beta ,\;\mathrm{and}\;\gamma$) exist in PLA depending on the crystallization conditions. The α-form is the most common crystal structure, in which polymer chains are suggested to form a helix conformation [213]. It can be formed under conventional melt and solution crystallization conditions (crystallization temperature (*T*_c_) > 120 °C) [209]. After being foamed, the crystalline phase of PLA was found to be mainly α-form [49]. Zhang et al. [214,215] reported that a disordered $\alpha^{\prime }$-form of PLA could be crystalized below 120 °C, which showed a similar helical chain conformation to the α-form, but was less compact. Similar findings were described by Pan et al. [216] for l-PLA with different MWs, whereby the $\alpha^{\prime }$-form could be produced at T_c_ below 100 °C, while, at T_c_ between 100 and 120 °C, both α- and $\alpha^{\prime }$- forms could be formed. Strain-induced $\alpha^{\prime }$-crystals and mesophase were found by Stoclet et al. [217] due to the structural rearrangement of PLA under tensile drawing. Puchalski et al. [218] investigated the formation of PLA crystals during the fiber-spinning process. At high draw ratios above 650%, the ordered α-crystal was developed, while the $\alpha^{\prime }$-crystal was found at lower draw ratio. Furthermore, the transformation from α- to β-crystal in l-PLA was observed during the solution-spinning process at high drawing temperature and/or high draw ratio by both Eling et al. [219] and Hoogsteen et al. [220]. Compared to chains in α-crystals, chains in the β-crystals exhibit more extended helical conformation [219]. The *T*_m_ of the β-crystal is about 10 K lower than that of the α-crystal, implying its lower thermal stability [220]. In addition, γ-crystal modification is a more ordered structure which is based on hexagonal packing, which can be obtained via epitaxial crystallization of PLA on hexamethylbenzene (HMB) [221]. 

PLA is known for its slow crystallization kinetics [33]. It is expected that PLA with moderately increased crystallinity favors the foaming process by enhancing PLA’s melt strength and viscoelastic behavior, such that cell coalescence and cell rupture can be reduced [49,122]. Nevertheless, Zhai et al. [33] observed that, when the crystallinity was too high by extending CO_2_ sorption time in the batch foaming process, foam expansion was inhibited due to the stiff PLA matrix. Generally, in the foaming process, crystallization of PLA is influenced by different factors, such as chemical modification, plasticization, thermal treatment, addition of nucleating agents, and extensional and shear deformation [33]; these factors are separately discussed below.

### 4.1. Influence of Chemical Modification

For linear PLA, *T*_g_ increases with MW and then reaches a constant value, which can be expected from the Fox–Flory equation, since chain mobility decreases with increasing MW [172,211,212]. In comparison to linear PLA, modified PLA with branched structures has a lower *T*_g_, which can be attributed to the higher free volume created by side chains [209]. Unlike *T*_g_, the *T*_m_ is generally less sensitive to branched structures [209]. Therefore, Mihai et al. [97] and Nofar et al. [222] observed only a little effect of the addition of CE on *T*_m_. Moreover, the cold crystallization temperature (*T*_cc_) is higher for linear PLA in comparison to branched PLA, due to the high chain mobility of linear PLA inhibiting the chains packing earlier [222]. With a higher amount of branching, *T*_cc_ decreases further due to the higher crystal nucleating potential of the branched or cross-linked structures [97,163,183,223]. Noteworthily, low-MW degradation products are generated during reactive extrusion, which can act as plasticizers. As a consequence, *T*_m_, *T*_g_, and *T*_cc_ of modified PLA decrease to some extent [124,125,183,187,191]. The crystallinity of modified PLA was generally reported to be reduced due to the restricted chain motion by both increased MW [209] and branching [97,187]. On the contrary, Nofar et al. [223] proposed that, in addition to the hindering effect on chain motion, crystallinity could also be affected positively if the chain end groups function as crystal nucleation sites, and both effects could suppress each other. 

### 4.2. Influence of Nucleating Agents

In general, the addition of nucleating agents speeds up the crystallization through a reduction of the energy barrier and results in a higher crystallinity. Various nucleating agents were applied for compact PLA, such as talc [224,225], carbon nanotubes [226], calcium carbonate, montmorillonite [227], etc. Additionally, enantiomeric chains of l-PLA and d-PLA can co-crystallize and form a stereocomplex [228]. Tsuji et al. [229] incorporated d-PLA as stereocomplex crystallites for l-PLA. The d-PLA with a nucleating effect accelerated crystallization and increased the number of l-PLA spherulites significantly. Brzeziński et al. [230] reviewed the recent development of PLA with functionalized carbon nanotubes and stereocomplexation, and suggested that their synergic effect could result in improved thermal and mechanical properties.

### 4.3. Influence of Plasticization

The most common used blowing agent CO_2_ has a strong plasticizing effect, which consequently influences the crystallization behavior of PLA (i.e., depression of *T*_g_ and *T*_m_) due to increased free volume and chain mobility, but the crystalline structure stays unaffected [231,232]. Furthermore, the crystallization rate and the final crystallinity change as well. As investigated by Takada et al. [232] using a high-pressure differential scanning calorimeter (HP-DSC), the crystallization rate was first accelerated by CO_2_ at lower temperature (crystal-growth-rate-controlled region) and then depressed at higher temperature (nucleation-controlled region). Nofar et al. [223] discussed the effect of CO_2_ pressure on PLA crystallinity based on experiments carried out in an HP-DSC. Here, CO_2_ at low pressure up to 15 bar facilitated the PLA chain movement and created more close-packed crystals and, therefore, higher crystallinity. However, at CO_2_ pressures above 15 bar, the final crystallinity of PLA decreased due to the hindered crystal growth by the large number of crystal nuclei. The addition of CO_2_ also showed a significant effect on the crystallization of PLA in foam extrusion. Mihai et al. [48] demonstrated that a higher amount of CO_2_ from 0 to 9 wt % added during foam extrusion generated a higher crystallinity of semi-crystalline PLA from 0 to 30%, while amorphous PLA with a high d-content remained amorphous even after foaming with CO_2_. 

### 4.4. Influence of Deformation

Furthermore, the crystallization rate of PLA can be considerably enhanced by extensional and shear deformation. Thus, in processes, where PLA is uniaxial or biaxial stretched, such as in foaming, in blow molding, or in melt spinning, strain-induced crystallization occurs, where crystallization is promoted by chain orientation and phase transformation [233,234]. This increase in chain orientation also leads to a significant crystallinity increase after processing [77,97]. In foam extrusion, PLA melt passes through a die, in which it undergoes sever shear deformation. Wang et al. [49] confirmed that the shear-induced crystallization happening in the die was governed during foam extrusion rather than extension. During foam cell growth, biaxial stretching is induced in cell walls, while uniaxial stretching happens in struts [116].

### 4.5. Influence of Thermal Treatment

In batch-foaming processes, PLA undergoes an isothermal treatment, i.e., annealing, under a certain temperature for a certain time to enable blowing agent saturation. Upon annealing, structural rearrangement happens as chain mobility increases. Thus, small and imperfect crystals change into more stable, more closely packed and more perfect crystalline structures, which is called crystal perfection, and a higher melting peak appears as a consequence [235]. The double peak of semi-crystalline polymers is extensively developed in bead foaming as it contributes to improved moldability and maintain the foam morphology. This is explained in detail in Section 5.4. In addition to the isothermal annealing process, non-isothermal treatment also influences the PLA crystallization greatly. Due to PLA’s slow crystallization kinetics, it turns out to be highly amorphous upon rapid cooling or quenching [119]. Upon decreasing cooling rate, the crystallization temperature increased, indicating that crystallization took place earlier [226]. Therefore, the cooling rate of PLA also needs to be taken into consideration after processing.

## 5. Processes

Polymer foaming can be carried out by batch processing (i.e., in an autoclave), foam extrusion, foam injection molding, or bead foaming. All methods were applied to PLA. Detailed explanations of the physical background were reviewed extensively before [236,237,238,239]. This review should give an overview of foam densities ρ and cell sizes achieved with PLA. The density is distinguished in terms of volume expansion rate (VER) as high (VER ≤ 4), medium (VER ≥ 4–10), and low (VER ≥ 10–50), as was done by a former review of Okolieocha et al. [236]. Assuming a raw density of 1.240 kg/m^3^ for PLA, this means a foam density above 310 kg/m^3^ is defined as high, while low foam densities are below 124 kg/m^3^, and medium densities can be found in between. Frequently used definitions to express the foam expansion are listed in Table 3. In the paragraphs, the data from literature are unified to absolute values in kg/m^3^ for comparison. 

### 5.1. Batch Foaming

**Figure 2:**
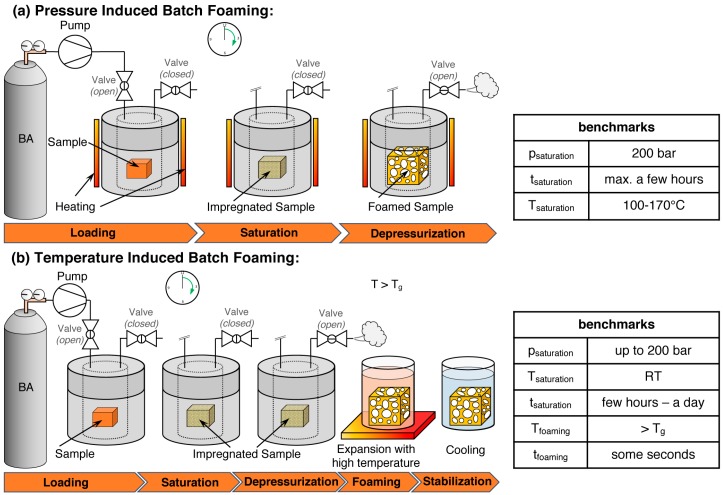
Principles of (**a**) pressure-induced batch foaming (at the top), and (**b**) temperature-induced batch foaming (at the bottom). For orientation, some benchmark parameters are given (note: for more detailed experimental set-up and parameters, please consult the literature).

Batch foaming is a discontinuous process conducted in an autoclave. As shown in Figure 2, it can be distinguished by the step that initiates the foaming as (i) pressure-induced batch and (ii) temperature-induced batch foaming. In both cases, the samples are saturated in a pressure vessel for a certain time. Then, by applying thermodynamic instability, foaming is induced. 

In the case of pressure-induced foaming, this is a pressure drop; by opening the outlet valve quickly, the pressure drops suddenly, and the heated polymer gets abruptly over-saturated and the previously solved gas cannot be retained by the polymer. Then, phase separation occurs and cell nucleation and growth take place, leading to the expansion of the sample with a porous structure. 

The saturation with the blowing agent during temperature-induced batch foaming is done at low temperatures and high pressures. The gas-loaded sample can be taken out of the autoclave without an immediate expansion. By immersing the saturated sample in hot media such as water [32,82,105], glycerin [86,240,241], or oil [242,243], foaming is initiated, since the applied temperature (above the *T*_g_) leads to (i) an increased chain mobility as the polymer gets softened, and (ii) a tremendous decrease in the solubility of the gas in the polymer. Again, this results in cell nucleation and growth. A cooling step ensures stabilization of the foam. 

In Figure 2, typical benchmark values for the process parameters are given. Both methods are mainly relevant for scientific research as it is a discontinuous process and rather small samples can be obtained. 

**Figure 3:**
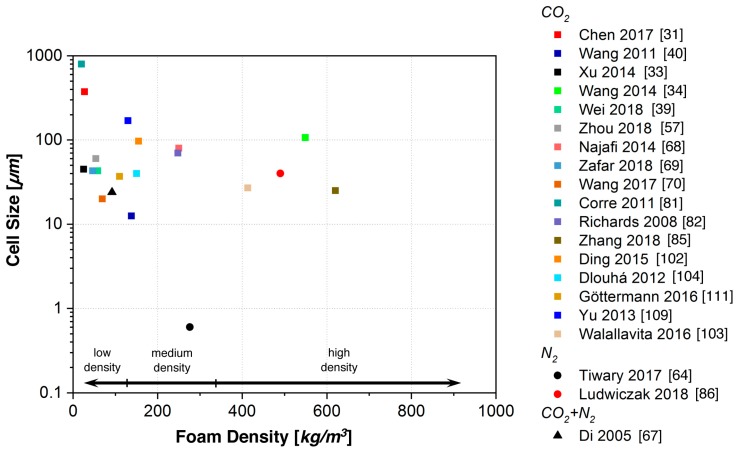
Overview of the literature on batch-foamed PLA (lowest density values with corresponding cell sizes within the given range were considered).

The achieved cell sizes and foam densities for batch-foamed PLA described in the literature (cf. Table 1) are summarized in Figure 3. Only a few reports exist using the temperature-induced method [32,82]. This method is usually applied to amorphous polymers because high crystallinity—that in the case of PLA can also be induced in the presence of the blowing agent during saturation—hinders a uniform cell nucleation and impedes the cell growth. In the approach of Wang et al., the samples were immersed in an ultrasonic irradiated water bath, and the expansion ratio and cell density increased, while cell sizes decreased. 

However, most works were conducted using the pressure-induced method. In the study of Corre et al. [81], the use of an epoxy-based chain extender was investigated and an enlarged foaming window was found. Similar works were carried out by Najafi et al. [68] and it was found that branching significantly sustained the cell uniformity and cell density because of the increased melt strength. 

Di et al. [67] foamed PLA modified with BDI and butanediol using a mixture of CO_2_ and nitrogen (20/80) as a blowing agent. Here, a significant reduction in density (down to 66 kg/m^3^) and cell size (24 µm) was achieved. According to the authors, the foamed structures blown with a CO_2_/nitrogen mixture were different from those blown with pure CO_2_, as the gas volume is higher [66]. 

The batch foam method is very sensitive to the temperature during foaming, as shown by Chen et al. [31]. Foaming PLA modified with an epoxy-based CE at a temperature of 144 °C resulted in a foam with 133 kg/m^3^ and a small cell size of 4 µm, while, at 152 °C, foams with a density of 27 kg/m^3^ and very large cells of 374 µm were obtained. This was attributed to the formation of higher-melting crystals that can form during the saturation at the lower temperature, which increase the density of cell nucleation and restrict the cell growth. This phenomenon is comprehensively described for PLA bead foams by Nofar et al. [116] and is further explained below (cf. Section 5.4).

A submicron-sized foam was achieved by Tiwary et al. [64] using nitrogen as a blowing agent. They addressed the viscosity as the main factor determining the cell size. With branched PLA, strain hardening was observed as beneficial to decrease cell sizes and increase cell densities. Here, the branching was achieved by a reactive modification, namely peroxide-initiated grafting of the multifunctional co-agent triallyl-trimesate (TAM). In pressure-induced batch foaming experiments, a foam with a void fraction of nearly 0.78 (approximately 270 kg/m^3^), a cell density of 10^11^ cells/cm^3^, and a cell size of 0.6 µm was achieved. 

### 5.2. Foam Extrusion

**Figure 4:**
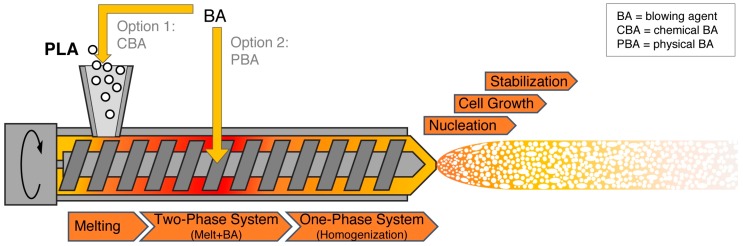
Principle of foam extrusion.

Unlike batch foaming, foam extrusion is a continuous process that is well established in industry. A sketch of the foam extrusion process with one extruder is shown in Figure 4. Combinations of two extruders are also common and this process is called tandem line. The polymer pellets are fed into the hopper and are conveyed by the screw(s). Due to heat and shear, the polymer is plastified. Blowing agents can be added into the hopper (in the case of chemical blowing agents (CBA)) or at injection points in the barrel (in the case of physical blowing agents (PBA)). A CBA is a substance that decomposes in certain conditions (i.e., high temperature) and releases gas(es). Usually, a solid residue will remain in the polymer which can act as the nucleating side. This self-nucleating effect can be more pronounced upon increasing CBA concentration [244]. However, PBAs (e.g., carbon dioxide or nitrogen) are more commonly used. Under the high pressure in the barrel, the polymer melt and blowing agent turn from a two-phase system into a homogeneous mixture. Following the direction of conveying, the gas-loaded melt is successively cooled down. Consequently, viscosity and pressure increase further. The gas-loaded melt exits the die and is subjected to ambient conditions, and a sudden pressure drop occurs. Here, cells are nucleated and start growing. Stabilization of the formed foam structure depends on the temperature. The extruded foams are limited in their geometry, which depends on the die shape (e.g., hole, slit, or ring die). The extrudate can be calibrated and cut prior to post-processing. Typical examples for extrusion foams are insulation boards or foamed packaging trays.

**Figure 5:**
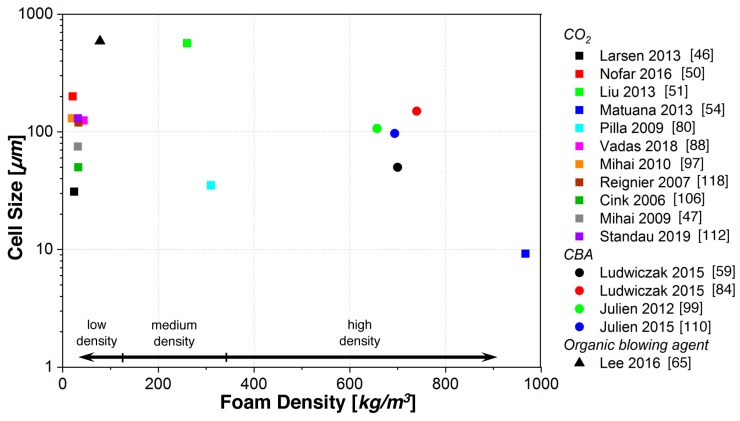
Overview of the literature on extrusion-foamed PLA (lowest density values with corresponding cell sizes within the given range were considered).

A conclusive visualization of the densities and cell sizes from works on extrusion foams is given in Figure 5. For PLA, rather lower expansion can be expected with chemical blowing agents because of the limited gas yield. Consequently, only a few reports were done, mainly with azodicarbonamide (ADC) [43,59,60,84,99,110]. Also, nitrogen is rarely applied as a blowing agent for PLA and rather less expansion was achieved [61]. Another work reports the use of a combination of CO_2_ and nitrogen resulting in foamed sheets with densities down to 350 kg/m^3^ [54]. Furthermore, some works with organic blowing agents such as iso-butane [65] or *n*-pentane [18] can be found, reporting foams with minimum densities of 78 and 38 kg/m^3^, respectively.

Most works were carried out with CO_2_ as a blowing agent. In the work of Matuana et al. [22,50], CO_2_ was used at the comparably low concentration of 5 wt %. Hence, the achieved expansion here was rather less with a void fraction of approximately 22 (970 kg/m^3^), but very small cell sizes below 10 µm were observed. However, usually more pronounced density reductions can be expected from higher concentrations of CO_2_, as reported in the literature. Systematic studies on how the CO_2_ concentration affects the expansion behavior were done by Reignier et al. [118] and also by Larsen and Neldin [46]. The authors stepwise increased the concentration of CO_2_ up to 10 wt % and recognized a significant drop in foam density down below 30 kg/m^3^ at around 8 wt %. High CO_2_ concentrations were also reported by other researchers to be necessary to achieve PLA foams with low density of around 20 to 30 kg/m^3^ [46,51,97,106,118] with corresponding cell sizes in the range of 30 to 800 µm. Thereby, PLA expansion is quite similar to extruded polystyrene. 

Interestingly, for CO_2_-based extrusion foams, low densities are reported in the literature even for neat PLA. Here, the addition of chemical modifiers does not necessarily lead to a further density reduction [46,97]. However, in many of the reports in literature, issues such as low melt strength and the occurrence of cell rupture or high open-cell contents are pointed out [46,47,118]. Chemical modifications are typically used in the foam extrusion process (cf. Table 1) because the foam structure and the resulting mechanical properties are mostly affected [46]. For example, Pilla et al. [80] reported a decreased cell size, a more uniform cell morphology, and higher expansion for PLA foamed with a multifunctional epoxy-based chain extender.

For foam extrusion, pressure drop rates of 1 to 10 GPa/s can be expected as shown in the work of Larsen and Neldin [46] depending on the geometry, e.g., die lengths. Here, lower-density foams were obtained from a shorter die which possessed a higher pressure drop rate.

An interesting study of Matuana et al. [50] focused on the understanding of the cell nucleation mechanisms of PLA foamed with CO_2_, examining the effect of processing temperature on the melt viscosity and the pressure drop rate. It could be shown that fewer cells are nucleated when PLA is processed at higher temperatures, as the decreased viscosity prevents a sufficient pressure increase, resulting in a lower pressure drop rate. Lower processing temperature leads to higher pressure drop rates and foams with smaller cell sizes and high cell-population densities. On the other hand, a higher processing temperature will lead to increased gas diffusivity, resulting in less gas to be solved in the polymer melt and, consequently, less nucleation and expansion. 

The crystallization behavior of PLA in a foam extrusion process was investigated by Tabatabaei and Park [62] with a special in situ visualization technique located in the die. It was shown that the crystallization kinetics were promoted by a strain-induced crystallization within the die. Furthermore, the effect of the strain rate was investigated. An increasing flow rate results in a higher strain rate and, consequently, crystallization is enhanced. If the number of formed crystallites increases, the cell density increases and higher expansions can be reached.

### 5.3. Foam Injection Molding (FIM)

**Figure 6:**
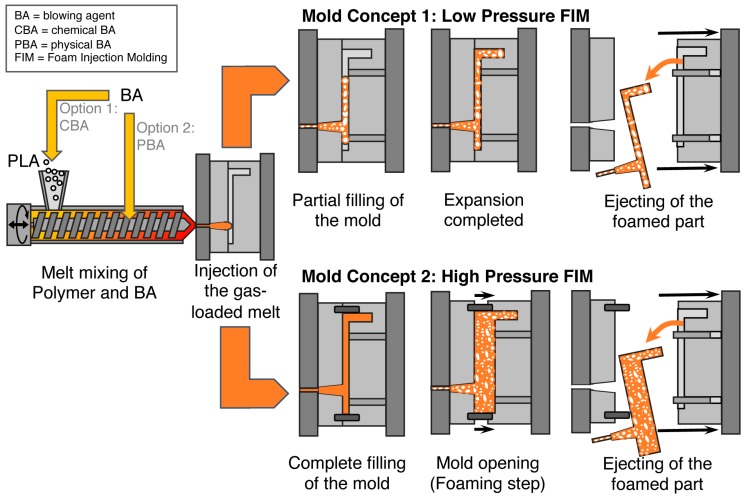
Principle of foam injection molding with the two optional mold concepts.

Generally, foam injection molding (FIM) is quite similar to injection molding, but is carried out with a blowing agent and requires some constructive features such as a special nozzle. Within this technique, several alternatives with different concepts for blowing agent incorporation and mold design exist, as can be seen in Figure 6. For incorporation of the blowing agent, two options are established. Either the blowing agent is dosed with the unmolten polymer in the hopper (cf. Figure 6, mold concept 1) or it is injected into the polymer melt in the barrel (cf. Figure 6, mold concept 2). In principle, the gas-loaded melt is conveyed by the screw toward a mold through a rotation movement. In addition to this rotational movement, the screw also moves backward, accumulating the gas-loaded melt at the tip, to inject it into the mold following a subsequent forward movement. Two mold concepts are in use, namely low- or high-pressure foam injection molding (FIM). 

In the case of low-pressure FIM (also referred as “short shot”), the mold is only filled partially with the gas-loaded melt, which exceeds a pressure drop instantly when being injected. Consequently, foaming happens immediately and the mold gets fully filled due to the occurring expansion. 

In contrast, during high-pressure FIM, the mold is completely filled under high pressure. Hence, possibly released gas would be solved again in the melt. Then, foaming is initiated by increasing the mold volume. Therefore, the mold is opened partially and the resulting pressure drop leads to expansion of the injected polymer melt. This method is also called “breathing mold” or “full shot”. 

The advantages of FIM are a better dimensional stability and less material consumption. Furthermore, longer flow paths and faster cycle times compared to usual injection molding can be realized due to the plasticization effect induced by the added blowing agent (i.e., the glass transition temperature decreases and the melt viscosity is reduced) [89]. A more detailed description can be found elsewhere [245]. 

**Figure 7:**
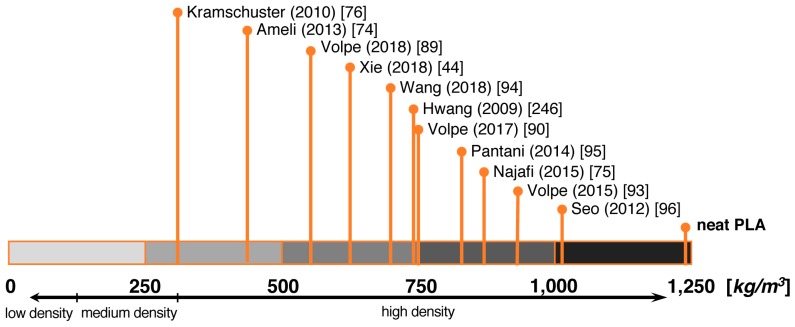
Literature overview of the (lowest) achieved densities in foam-injection-molded PLA.

Both chemical and physical blowing agent are used for FIM. In the examined literature, only a few publications on the foam injection molding of PLA with CBAs can be found [75,96]. Common physical blowing agents used for foam injection molding of PLA include N_2_ [74,90,92] and CO_2_ [58,76,246]. As far as the data given in the publications about injection-foamed PLA (cf. Table 1), the lowest achieved densities are summarized in Figure 7. It is striking that almost all works were carried out with unmodified PLA with lower molecular weights most likely to enable sufficient flow during the injection. 

The works carried out with chemical blowing agents reveal a rather low density reduction. Najafi et al. [75] used activated azodicarbonamide (ADC) as a blowing agent and achieved a relative density of 0.83 (approximately 1030 kg/m^3^) for neat (linear) PLA with a non-uniform cellular structure with average cell sizes of 68 µm. Due to the addition of nano-clay, the relative density was decreased to 0.77 (approximately 955 kg/m^3^). Cell sizes were also decreased to 35 µm. Chemically modified PLA with long-chain branches was also foamed with nano-clay, resulting in a relative density of 0.7 (approximately 870 kg/m^3^) and a reduced cell size of 29 µm. 

Seo et al. [96] compared injection-molded foams blown by PBA (nitrogen), CBA (ADC), and combinations thereof. Here, the combination was found to be most efficient in terms of reducing the cell size by a factor of two down to 50 µm. However, for PLA the highest foaming rate was reported as 14.1%, which converts to a rather high density of approximately 1060 kg/m^3^.

Intensive work on FIM of PLA with nitrogen was carried out by the group of Pantani and Volpe [89,90,92,93,95]. In an early work, they pointed out that higher injection flow rates resulted in a more homogeneous cell morphology and density reduction along the flow path. A higher mold temperature and the addition of talc resulted in parts with a density reduction up to 33% (approximately 830 kg/m^3^), very narrow cell size distributions, and increased crystallinity. [95]

Most efficient density reductions of PLA were achieved with high-pressure FIM. As shown by Xie et al. [44], a rather high void fraction of 50% and higher cell density were reached with a mold opening of 2 mm. The rate of mold opening was investigated, and significant effects on cell structure were noted. A low mold opening rate led to decreased cell sizes, higher cell densities, and smoother surfaces. The impact resistance of the foamed parts was mainly influenced by the void fraction. The effect of mold opening on the morphology and mechanical properties for PLA blown with nitrogen was also investigated by Volpe et al. [89]. A wider mold opening led to a higher density reduction, as the same amount of injected material filled a larger volume. A density reduction of more than 50% was reported. Furthermore, it was stated that a higher crystallinity and a more homogeneous morphology in the foam-injected parts results in a higher flexural strength. With mold opening, the highest reported void fraction of 65% (approximately 430 kg/m^3^) was achieved by Ameli et al. [74]. Here, a fourfold increase in flexural rigidity and a 15% higher specific impact resistance were reported. With nano-clay, cell sizes down to 50 µm were achieved. 

Kramschuster et al. [76] succeeded in producing PLA with the highest porosity of 75% (approximately 310 kg/m^3^) in the FIM process of PLA with a special approach. Prior to FIM, PLA was compounded with polyvinyl alcohol (PVA) and a relative high amount of 60 vol.% NaCl. CO_2_ was used as a blowing agent and plasticizer to enable the processability even at this high salt loading. The molded parts were leached with deionized water to remove the salt and PVA. Hence, the density depended on the leaching time. Highest porosity was achieved after 18 h. However, NaCl could partially remain in the samples, and interconnected pores with a relatively large diameter of 200 µm were reported. 

### 5.4. Bead Foaming

Currently, the bead foams with the biggest market shares are made from polystyrene (expandable PS (EPS)), which is mainly used for packaging and building insulation, and polypropylene (expanded PP (EPP)), which can be often found in automotive applications. Advantages of bead foams are that complex shapes and low densities can be combined [237]. Currently, other polymers such as thermoplastic polyurethane (TPU), polybutylene therephtalate (PBT), PET, or PLA [237,247] received attention in research as they possess enhanced properties. In general, bead foaming is a two-step process as described below.

**Figure 8:**
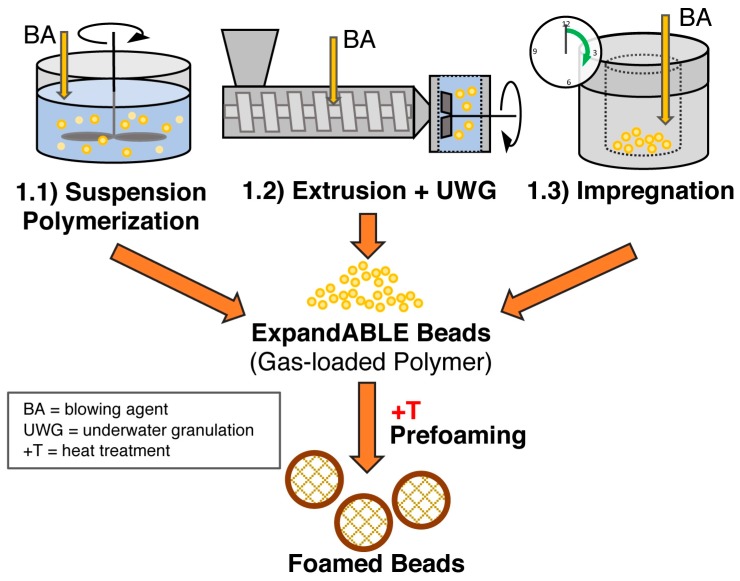
Overview of different methods to produce expandable bead foams.

**Figure 9:**
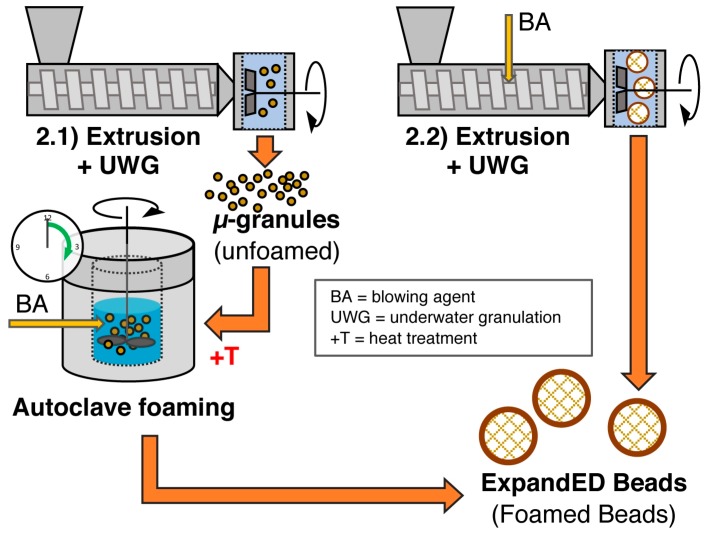
Overview of different methods to produce expanded bead foams.

Firstly, single foamed beads have to be produced. Several routes are possible depending on T_g_ and crystallinity of the polymer, resulting in expandable (T_g_ > room temperature, amorphous) and already expanded beads (T_g_ < room temperature, semi-crystalline). In Figure 8, the methods to achieve expandable beads are summarized. The most prominent example for these expandable beads is EPS which is usually produced with method 1.1 (suspension polymerization) or method 1.2 (extrusion with underwater granulation (UWG)). In Figure 9, the methods to obtain expanded bead foams are demonstrated. EPP—the most-relevant expanded bead foam—is mainly prepared using method 2.1 (autoclave foaming) and, rarely, using method 2.1 (extrusion with UWG). Interestingly, because of the complex crystallization behavior of PLA, both expandable and expanded bead foams can be obtained. Secondly, these foamed beads have to be consolidated to a final part. Usually, this is done in a so-called steam chest molding machine (SCM), the process of which is explained below (cf. Figure 12). In the literature, the final part consolidation is also named the fusion, welding, or sintering process.

### 5.5. Method 1.1—Suspension Polymerization with Organic Blowing Agents

Expandable polystyrene (EPS)—the first polymeric bead foam in history—was made in the 1950s by suspension polymerization with an added organic blowing agent [248]. It must be emphasized that, in this matter, gas-loaded particles are obtained. This is beneficial for transportation since the unexpanded beads have low specific volume. Depending on the storage conditions, the shelf life of the gas-loaded particles can be a month or longer. Prior to further processing, they have to be pre-foamed by heat treatment, usually with steam. For EPS, very low densities below 20 kg/m^3^ can be achieved [249]. An approach to incorporate clay during the suspension polymerization of PS and foaming (lowest density 30 kg/m^3^) was described by Shen et al. [250]. Nevertheless, the incorporation of additives is much more convenient with the methods described below. However, a PLA bead foam derived from method 1.1 is yet to be described in the literature.

### 5.6. Method 1.2—Extrusion with Blowing Agent Combined with UWG and Suppressed Expansion

BASF SE produces a bead foam from a PLA/PBS blend following method 1.2 [20,21]. Pentane-loaded microgranules are continuously produced with the addition of a multifunctional epoxy-based CE by extrusion with UWG. To prevent foaming during the extrusion, the water pressure has to be above the vapor pressure of the blowing agent (i.e., 10.1 bar for pentane at 125 °C [237]). Pre-foaming of the obtained amorphous and gas-loaded microgranules is done with hot steam, resulting in low-density beads very similar to EPS.

### 5.7. Method 1.3—Impregnation

For neat PLA bead foams, method 1.3 is mainly used. Patents from the company JSP Corp. [23,24] and the Biopolymer Network Ltd. (BPN) [73,105], as well as the work of Parker et al. [251], describe the procedure of impregnating the polymer at low temperatures. However, significant desorption of the impregnated beads of 50 to 75% loss even at very low temperatures of −20 °C was noted, which is a drawback compared to gas-loaded microgranules used for EPS production, which can be stored for several weeks when pentane is used as a blowing agent [249]. The impregnation is followed by pre-foaming carried out in hot water or with a mixture of steam and hot air, resulting in low-density bead foams. 

An enhanced approach was followed by the company Synbra, as can be found in their patent [19]. After impregnation and pre-foaming, an additional coating of polyvinyl acetate solution is used as a sticking agent to improve the fusion behavior of the beads, i.e., the beads are more or less glued together with it. Thus, increased compression and breaking strength and less shrinkage were obtained for the welded parts.

### 5.8. Method 2.1—Extrusion with UWG and Autoclave Foaming

**Figure 10:**
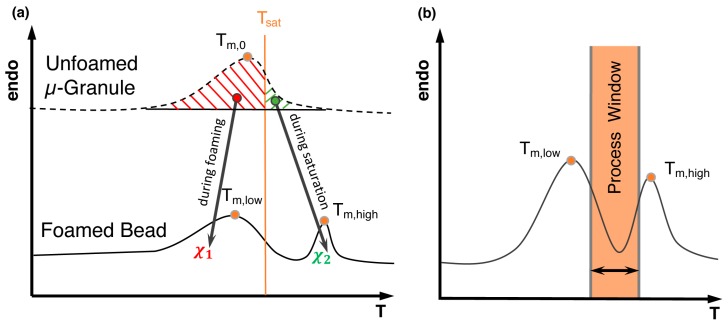
Sketch of (**a**) double melt peak development, and (**b**) process window for fusion in steam chest molding based on References [237,259].

It is known for most of the semi-crystalline polymers that it is possible to create multiple melting peaks as already identified by Harrison [252]. Different crystal sizes [253], different crystal structures [254,255,256], and lamella thickening or rearrangement to higher order (so-called crystal perfection) during heating or isothermal phases [257] were acknowledged as prospects to create a double melting peak. For PP, the creation of the double melting peak sensitively depends on the temperature, as shown by Hingmann et al. [258]. The phenomenon of a double melting peak is used to ensure the fusion of polypropylene-bead foams obtained from the autoclave foaming process [237]. A good description of the influence of processing parameters can be found in the work of Nofar et al. [259]. Usually, the microgranules are saturated at a temperature close to the polymer’s melting point in a water-filled autoclave, and they are stirred while CO_2_ is applied at high pressures. Foaming is initiated by a sudden pressure drop (i.e., opening of a valve at the bottom of the autoclave). During the isothermal saturation phase, the unmolten crystals with increased chain mobility are able to rearrange into a higher order (crystal perfection) leading to a novel, higher melting peak. During the foaming, the formerly molten crystals turn back into their original state, forming the lower melting peak which is located at the original melting point. This is schematically shown in Figure 10. The processing window of the fusion (steam chest molding) is usually between the two melting peaks.

In further works, Nofar et al. [115,116] transferred this double melting peak concept to PLA. PLA is highly sensitive to hydrolysis and degrades during the saturation process. Thus, the PLA was modified with multifunctional epoxy-based CE, and silicon oil was added as a hydrophobic surfactant into the suspension media to prevent a too strong impact of the degradation on the foaming. Even though the gel permeation chromatography (GPC) measurement revealed a decrease in MW after saturation at an elevated temperature, foaming was still successful. However, the reduction of MW consequently led to lower melt strength. Hence, a high open-cell content (up to 90%) was noted. This could be reduced by a shorter saturation time. It was found that hydrolysis was more impacted by the time than the temperature. Using this method, volume expansion ratios of 5–40 (approximately 250–30 kg/m^3^) were reported [260]. Additionally, the perfected crystals could act as heterogeneous cell nucleators, resulting in fine cells down to 60 µm. 

Furthermore, bead foaming with a stirring autoclave (method 2.1) was also done by Tang et al. [261] for PP/PLA blends with n-pentane as a blowing agent. The PLA was added with a content of 30 wt.% as it possesses a significantly higher solubility of the blowing agent, which was attributed to its lower crystallinity. With pentane, the plasticizing effect is very pronounced, resulting in a rather broad foaming window at lower temperatures (85 to 99 °C). Bead foams with high expansion ratios up to of 44.4 (approximately <30 kg/m^3^) and high cell densities were achieved. Here, no double melting peak was reported and fusion trials were not conducted.

### 5.9. Method 2.2—Extrusion with Blowing Agent Combined with UWG and Expansion

A continuous method to produce foamed particles in one step is method 2.2, which was applied to PLA as described by a patent from the company Sekisui Plastics Co., Ltd. [26]. PLA with low d-content from Unitika is processed with a single screw extruder with an attached underwater granulator. Here, butane is used as a blowing agent. Foamed particles with diameters up to 3.6 mm and a density of 48 kg/m^3^ are reported. 

**Figure 11:**
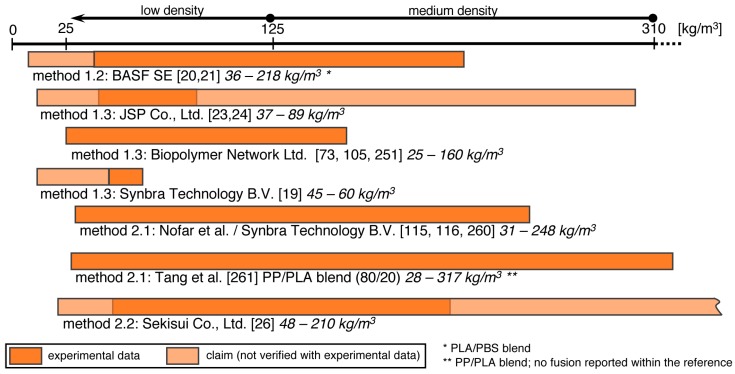
Literature overview of achievable density ranges for expandable PLA (EPLA).

In Figure 11, the achieved densities from the above-described PLA bead foams are summarized. The reported densities from experiments and examples are rather low (marked in orange). In patents, lower and higher densities are claimed but not proven with experimental data (marked in light-orange).

### 5.10. Steam Chest Molding

**Figure 12:**
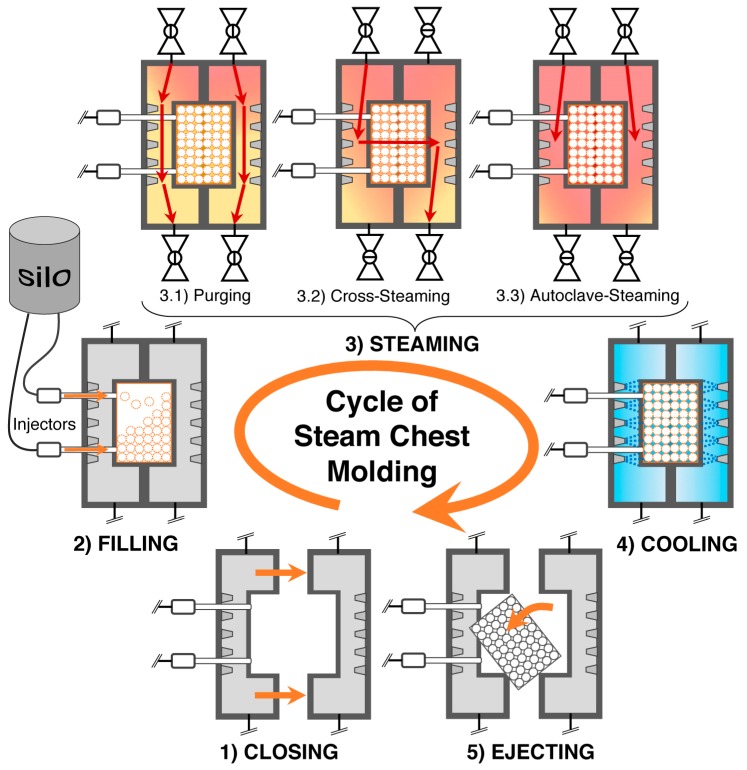
Process steps of the steam chest molding (see also Reference [237]).

The final part consolidation is classically done with hot steam in a so-called steam chest molding machine. Steps of this process are illustrated in Figure 12. The review of Raps et al. [237] is recommended for further information. 

Step 1 (Closing): Firstly, the mold is closed. The mold defines the shape of the final part. Steam nozzles are placed in the walls to ensure steam can come into the mold from the steam chamber during the later processing steps. 

Step 2 (Filling): Then, the closed mold is filled with (pre)foamed beads, which is done with special injectors usually operated by pressured air. 

Step 3 (Steaming): The steaming takes place in several sub-steps. Heating of the mold is done by purging with steam while keeping all valves opened, allowing the steam to remove the air between the beads. Then, during cross-steaming, one of the inlet valves is opened, while the opposite outlet valve is closed. Thus, the steam goes through the mold. This repeats with changing valve positions and the mold is steamed from the other side to ensure uniform welding in the whole part. A skin on the part is created while autoclave steaming with closed outlet valves.

Step 4 (Cooling): Afterward, steaming cold water is injected. This is necessary to cool down the mold and prevent further dimensional changes. 

Step 5 (Ejecting): After opening the mold, the final part is ejected by pressured air or mechanical ejectors, and the cycle from steps 1 to 5 can be repeated immediately. 

The degree of fusion has strong influence on the final part properties, such as compression [262], tensile [263], and fracture behavior [264]. Rossacci and Shivkumar [265,266] gave basic insights into the influence of the fusion quality on the properties. The degree of fusion can be judged by image analysis of the fractured surface of a tensile tested sample. If the failure occurs dominantly through the bead, i.e., so-called trans- or intra-bead fracture, a good fusion is achieved. It was pointed out that thick parts differ regarding fusion quality from the surface and interior. Also, with improved bead fusion, the number of voids is reduced.

Welded EPLA was compared regarding its properties with EPS by Parker et al. [251], revealing the same thermal conductivity of 0.03 W/mK at densities between 25 and 30 kg/m^3^. Furthermore, it could be shown that the mechanical performance in the case of compression and shear behavior was equivalent to EPS. Also, the mechanical properties linearly depended on the density. The compression and the breaking strength were also evaluated for the commercially available material from Synbra B.V. in their patent [19], albeit in qualitative terms. Information about flexural strength can be found in the patent of JSP Corp. [23], wherein the highest flexural strength of 1.84 MPa was measured at a density of 96 kg/m^3^.

Nofar et al. [115] even state that EPLA can be a suitable replacement for EPP, as the Young’s moduli and tensile strengths of EPLA with a double melting peak (method 2.1) are similar to those of EPP. Furthermore, strong inter-bead sintering characteristics were found, indicating a good fusion.

PLA bead foams are often referred to as so-called “drop-in” solutions for current EPS fabricators because the processing itself, the process conditions, and the properties are quite similar [251]. As usual, molding of the beads takes place in a closed mold [20] and steam is applied. Steaming times of several seconds are reported [23,105,115], allowing cycle times comparable to those for the standard bead foams. Steam temperatures up to 100 °C [19,105] or pressures of 2 bar [19] and 2.5 bar [115] can be found in literature. However, depending on the abovementioned production method, beads with high crystallinities can require higher steam temperatures of up to approximately 160 °C [23]. Part densities can slightly be higher than the density of the sole beads, and steaming and cooling conditions have to be adjusted carefully to ensure good fusion quality and to avoid shrinkage or warpage [19,23]. Alternatively, welding can also be performed with hot water (95 °C) as described by a patent of Sekisui Plastics Co., Ltd. [26].

Meanwhile, steamless alternatives are also emerging, involving highly dynamical contact heat, radiation, and coating with suitable additives [267]. Hence, following this trend, the impact of possible hydrolytic degradation could be reduced.

## 6. Trends and Perspectives

PLA foams received a lot of attention in scientific research. On one hand, PLA possesses properties that are very useful for certain processes and applications and, on the other hand, these properties need to be improved. These properties include the favorable biodegradability and biocompatibility, but also the low thermal stability and poor fire properties which need improvement. Here, a possibly imperfect summary about future focal points is given.

### 6.1. Biodegradability

Biodegradation is a process in which a biodegradable polymer is degraded by microbial organisms through metabolic or enzymatic processes. The complex molecules break down into smaller molecules and can be metabolized by microorganisms to water, carbon dioxide, and humus [268]. In nature, the degradation is often induced by thermal activation, hydrolysis, biological activity, oxidation, photolysis, or radiolysis. This makes the environmental degradation very complex, because of the coexistence of biotic and non-biotic processes, which can also take place simultaneously. In the case of PLA, degrading microorganisms are not widely distributed in the natural environment. Thus, PLA is not very susceptible to microbial attack in the natural environment and, consequently, not suitable for home composting processes [7,269].

The biodegradability of PLA depends on the environment to which it is exposed. PLA initially degrades via hydrolysis in human or animal bodies. Soluble oligomers are formed that can be metabolized by cells. If disposed in the environment, PLA hydrolyzes into low-molecular-weight oligomers that are then mineralized into CO_2_ and H_2_O by the present microorganism in the environment. Because of the small number of microorganisms that mineralize PLA, the degradation in soil is very slow. In a composting environment (45–60 days at 50 °C [269]), it hydrolyzes into small molecules such as oligomers, dimers, and monomers, which can be mineralized into CO_2_ and H_2_O by the microorganism in the compost in a much shorter time frame. In addition to microorganisms, enzymes also play a significant role in degradation processes. The enzymatic degradation via hydrolysis is a two-step process. Firstly, the enzymes adsorb onto the surface of the polymer; then, the hydrolysis of the ester bonds takes place.

In addition to the environmental conditions, there are several other factors that can affect the biodegradability of PLA, such as molecular weight (distribution), crystallinity, and surface properties [269]. In general, the biodegradation is slower with increasing molecular weight [269]. An increased crystallization is more resistant to degradation. While the biodegradation of compact PLA or PLA blends is investigated intensively, less literature is available on the biodegradation of foamed PLA. However, commercial products that are biodegradable according to standards are available [21].

### 6.2. Medicine

In the medical sector, promising applications for foamed PLA products can be found, such as tissue engineering or as drug release components. PLA is often used in bone or cartilage tissue engineering in the form of scaffolds. These polymers degrade in vivo via hydrolysis of their ester bonds and have a wide range of mechanical and physical properties. The following essential properties for scaffolds and the material have to be fulfilled [270,271]: (i) biocompatible, (ii) biodegradable or capable of being remodeled, (iii) should biodegrade in tune with the repair or regeneration process, (iv) very porous, (v) highly permeable to allow proper diffusion, (vi) have an optimal pore size, (vii) possess adequate mechanical properties, (viii) provide a surface for cell attachment, (ix) encourage the formation of extracellular matrix, and (x) should possess the ability to carry biomolecular signals such as growth factors.

The idea behind tissue engineering is to shape scaffolds into structures that mimic specific tissues or organs, and to load the scaffolds with living cells and nutrients. These are implanted afterward to replace diseased or damaged organs without the need to retrieve the scaffolds. To fabricate these tissue-engineering scaffolds, different methods can be used, such as fiber bonding, solvent casting, and gas foaming with particulate leaching or phase separation. The problem in almost all of the existing methods is that organic solvents are required, which may can reduce the ability for biological cells to form new tissue [271].

### 6.3. Thermal Properties

The T_g_ of PLA is about 55 °C, which is equal to a low heat resistance, consequently limiting its application. Also, the slow crystallization kinetics are adverse in this context. To overcome this issue, some works were carried out on the stereocomplex crystallization of PLA resulting in higher glass transition and melting temperatures [91,272,273]. In the work of Xue et al. [91], it could be shown for batch-foamed PLA that an increasing d-PLA content decreases shrinkage in boiling water.

However, because of its high price, the addition of d-PLA is not favorable. Another way to increase the heat resistance would be to enhance its crystallization kinetics, which can be induced—as described before—in the presence of a plasticizing blowing agent and by applying biaxial stress during the expansion or by annealing [6,274,275,276]. For extrusion foams, an increase in heat resistance up to 99 °C with increasing crystallinity can be found [18]. Also, for bead foams, the high obtained crystallinity (50 to 60%) results in a high heat resistance, as reported in the patent of Sekisui Co., Ltd. [26]. Here, welded PLA bead foams were stored in an oven at 120 °C for 22 h, and only minor dimensional changes of less than 1% were noted.

Lee et al. [65] introduced another approach for extrusion-foamed products with increased heat distortion temperature. Within their work, they laminated a compact highly crystalline PLA film on a low-density foamed PLA sheet, and created thermoformed structures that could withstand elevated service temperatures comparable to PS foams.

### 6.4. Flame Retardancy

A lot of applications (e.g., electronics, construction, and automotive) have high requirements concerning flammability and dripping combustions, which are not fulfilled by PLA. Yet, only few attempts regarding the improvement of flame retardancy were made for compact and foamed PLA.

Zhu and coworkers [277] reported an improvement for the flame retardancy of compact PLA using a synergistic mixture of expandable graphite (EG) together with ammonium polyphosphate (APP). With 15% of this intumescent flame retardant (APP/EG = 3:1), the Limiting Oxygen Index (LOI) was increased from 22 to 36.5 and UL-94-V-0 classification was reached. The same burning behavior was described by Ke et al. [278] using 30% of a mixture (3:2) of a novel hyperbranched polyamine charring agent (HPCA) together with APP. Good flame retardancy was also reached with synergistic combinations of aluminum hypophosphite and expanded graphite by Tang et al. [279], obtaining high LOI values, UL-94-V-0 classification, and anti-dripping effects.

The group of Zhai did some work on improving flame retardancy of foamed PLA with a phosphorous-containing flame retardant, and starch [280] or graphene [281] as a charring agent. Foaming was carried out with the batch method, and maximum expansion ratios of up to 17.5% (approximately 70 kg/m^3^) were possible. The LOI could be significantly increased, UL-94-V-0 classification could be realized, and anti-dripping effects were shown.

The incorporation of a bio-based flame retardant into a PLA extrusion foam was done by Vadas et al. [88]. Here, a combination of flame-retardant-treated cellulose (surface treatment with diammonium phosphate and boric acid) as a bio-based charring agent and APP as an intumescent flame retardant was used to reduce the flammability of PLA foams. A multifunctional epoxy-based chain extender was used and, even at high additive loadings, a significant expansion with void fractions above 90% (<124 kg/m^3^) was possible with CO_2_ as a blowing agent. Excellent flame retardancy (UL-94 V-0 and LOI of 31.5%) was achieved with an additive content smaller than 20%. Furthermore, it was found that the flame-retardant synergism was less pronounced in the expanded foams compared to the compact materials most likely because of (i) a distinctly enlarged contact surface, and (ii) a decreased volume concentration (i.e., dilution) of the flame retardant [280,281].

## 7. Conclusions

In this review, the strategies for improving the foaming behavior of PLA, with regards to chemical modification and processing techniques, were studied. For almost two decades, scientific research focused on foaming of the bio-based and biodegradable polymer PLA, which is often referred to as a good alternative to polystyrene. However, like many other polyesters, PLA possesses a rather low melt strength, which is the main challenge for foaming. Therefore, a huge variety of chemical modifiers for melt modification were investigated to overcome this issue. Chemical modifications increase the molecular weight and can induce structural changes (i.e., branching and/or cross-linking) of PLA, thereby significantly changing the rheological behavior. Here, the commonly used chemical modifiers and their effects on the rheological properties of PLA were reviewed. Depending on their types and the amount added, the viscosity and the melt strength increase, and strain hardening, which is beneficial for expansion processes, can be induced. Thus, with the incorporation of chemical modifiers, an improvement in the foam morphology (smaller cell sizes, higher cell density) and expansion can be expected.

By now, PLA foams made using all common methods can be found in the literature (i.e., batch foaming, foam extrusion, foam injection molding, and bead foaming). Meanwhile, PLA foams are already successfully applied industrially. PLA exhibits a complex crystallization behavior which is additionally influenced during the foaming process by various factors, such as plasticization by the blowing agents, deformation of the PLA melt, thermal treatment in the process, and the addition of chemical modifiers and nucleating agents. One consequence of this is that very different bead-foaming techniques, which are normally restricted to either amorphous or semi-crystalline polymers, can be applied to PLA as it can exhibit both characteristics. Thus, low-density PLA bead foams with overall properties very similar to EPS can be obtained.

The low temperature resistance of PLA foams is challenging for most applications. Approaches to overcome this mainly include the control of their crystallinity. Also, current works are attempting to improve the burning behavior of PLA foams. In addition to the improvements in PLA foam morphology and the resulting mechanical properties, biodegradability optimization and functionalization of PLA foams will govern more interest in future research. PLA foam with improved flame retardancy, thermal resistance, and controlled biodegradation behavior would widen the applications to a large extent and, thus, should be further investigated.

## Figures and Tables

**Table 1:** Literature overview of polylactide (PLA) grades (NatureWorks LLC) used for foaming (blends with other polymers were not considered). Please note: the original purpose of the grade as recommended by the supplier can be found in italic letters above the grade notation.

PLA Grade (NatureWorks)	Foamed		d-Content
	Neat	Chemically Modified	(%)
*Extrusion and thermoforming*			
2002 D	A [30,31,32,33,34,35,36,37,38,39,40], F [41,42,43,44,45], X [43,46,47,48,49,50,51,52,53,54,55]	A [31,35,36], X [46,47,52,55],	4.0–4.3 [32,37,41,44,48,49,55,56]
2003 D	A [57], F [58], X [59,60,61,62]	A [63], X [59,60]	4.3 [57]
2500 HP	A [64]	A [64]	0.4 [65]
*Injection molding*			
3000 D	A [66,67]	A [67]	N/A
3001 D	A [68,69,70,71,72], B [73], F [74,75,76,77,78,79], X [80]	A [68,70], F [75], X [80]	1.4–1.5 [71,76,78]
3051 D	A [81,82,83], X [84]	A [81,83], X [18]	4–4.15 [18,81]
3052 D	A [85,86], X [59,87]	X [59,87,88]	4 [85,87]
3251 D	A [64], F [89,90], X [46]	A [64]	1.4 [89]
*Films and cards*			
4032 D	A [91], F [89,90,92,93,94,95,96], X [48,97,98,99]	X [18,97,100]	1.4–2.0 [18,48,56,89,101]
4060 D	A [102,103,104], B [73,105], X [106], F [107]	B [19], X [106]	12–12.3 [56,106]
*Fibers and nonwovens*			
6300 D	X [106,108]		9.5 [106], 9.85 [108]
*Blow molding*			
7000 D	A [109], X [99,110]	A [109], X [110]	6.4 [110]
7001 D	A [111], X [111,112]	A [111], X [111,112]	4.4 +/− 0.5 [113]
*Foaming*			
8051 D	X [49], A [71,114]	A [71,114], B [20,21,115,116], X [49]	4.2–4.6 [49,71,114,115]
8052 D	A [117], X [46]	A [117], X [46,65]	4.7 [65]
8300 D		X [106]	11 [106]
8302 D	A [71], X [48,50,97,118]	X [97]	9.85–10.1 [48,71,118]

A = autoclave foam, B = bead foam, F = foam injection molding, X = extrusion foam.

**Table 2:** Overview of commonly used chain extenders (CEs) from the literature used for PLA melt modification sorted by their functional groups (Please note: references for foams from chemically modified PLA are marked in **bold**).

Type	Functional Group	Chemical Modifier	Reference
Epoxide	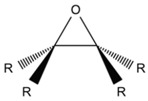	Multifunctional epoxy-based oligomer	[**18**,**31**,**35**,**46**,**49**,**52**,**59**,**64**,**65**,**68**,**70**,**71**,**75**,**80**,**81**,**83**,**87**,**88**,**97**,^101^,**110**,**112**,**114**,**115**,**116**,**117**,^121^,^123^,^134^,^135^,^136^,^142^,^143^,^144^,^145^,^146^,^147^,^148^,^149^,^150^,^151^,^152^,^153^,^154^,^155^,^156^,^157^]
Isocyanate	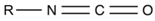	1,4-butane diisocyanate (BDI)	[**67**]
		1,6-hexamethylene diisocyanate (HDI)	[101,121,158]
		4,4-methylene diphenyl diisocyanate (MDI)	[127,159,160]
Anhydride	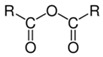	Pyromellitic dianhydride (PMDA)	[**36**,^101^,**109**,^143^,^161^,^162^,^163^,^164^,^165^]
Oxazoline	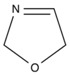	1,3-bisoxazoline	[121]
		1,4-phenylene-bis-oxazoline	[164]
		2,2-bis(2-oxazoline)	[162]
		Not specified	[109]
Carbodiimide (CDI)	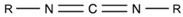	CDI	[166,167]
		Polycarbodiimide (PCDI)	[149,168,169]
		Bis(2,6-diisopropylphenyl) carbodiimide (BDICDI)	[166,170]
Phosphite	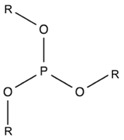	Tris(nonyl-phenyl) phosphite (TNPP)	[149,171,172,173,174]
		Triphenylphosphite (TPP)	[175,176]

**Table 3:** Overview of terminologies to express foam expansion frequently used in the literature.

Volume Expansion Rate (VER) (-)	Void Fraction ($V_{f}$), Degree of Foaming (-)	Density Reduction (DR), Foaming Ratio (%)	Relative Density (RD), Specific Gravity (-)
$VER=\frac{\rho_{polymer}}{\rho_{foam}}$	$V_{f}=1-\frac{\rho_{foam}}{\rho_{polymer}}$	$DR=\left(1-\frac{\rho_{foam}}{\rho_{polymer}}\right)\times 100=V_{f}\times 100$	$RD=\frac{\rho_{foam}}{\rho_{polymer}}=\frac{1}{VER}$
